# Improve sample preparation process for miRNA isolation from the culture cells by using silica fiber membrane

**DOI:** 10.1038/s41598-020-78202-8

**Published:** 2020-12-03

**Authors:** Wen-Pin Hu, Yu-Chi Chen, Wen-Yih Chen

**Affiliations:** 1 Department of Bioinformatics and Medical Engineering, Asia University, Taichung, 41354 Taiwan; 2 Department of Chemical and Materials Engineering, National Central University, Jhong-Li, No. 300, Zhongda Rd., Zhongli District, Taoyuan City, 32001 Taiwan

**Keywords:** Isolation, separation and purification, RNA

## Abstract

In clinical applications of miRNAs, the purity and quality of the testing samples are very critical, especially the obtained tissue sample volume is limited. If the extracted miRNAs are contaminated or different in quality before analysis, it will increase the variance of the analysis result and make the medical information judgment incorrect and cannot be portable. Herein, we improved the commercially extraction kit by realizing the fundamental mechanism and hoped to serve finding optimal procedures for increasing the recovery of miRNAs extracted from cultured cells. In the adsorption process, the factors, like increasing the ethanol concentration or adding Ca^2+^, could influence the RNA adsorption were investigated. For the elution process, the effect caused by raising the elution temperature and raising the pH value of elution buffer was studied. Finally, the conditions for miRNA extraction are optimal modified by using a 65% (v/v) solution of ethanol in the adsorption process, and using TE buffer with the pH value of 8.0 and raising the temperature to 55 °C in the elution. According to the quantified results, the improved extraction kit can promote the recovery of endogenous miR-21 by about 6 times by using the optimal extraction conditions comparing with the miRNeasy Mini Kit.

## Introduction

MicroRNAs (miRNAs) are noncoding RNA molecules with short lengths (about 18–22 nt), which play major roles in the posttranscriptional regulation of protein expression. MiRNAs have been found to be associated with a wide range of human diseases, like tumors and cancers, cardiovascular diseases, neurodegenerative diseases, leukemia, and so on^1,2^. Therefore, many studies suggest that miRNA expression profiles can be diagnostic and prognostic biomarkers for diseases. The critical point for developing precision medicine has to rely on the individual’s -omics profile. Some biomedical analysis techniques, including quantitative real-time polymerase chain reaction (qPCR), microarray, and next-generation sequencing (NGS), are commonly used to obtain genetic data. During the analysis process, sample collection and storage, and nucleic acid extraction and detection are also included in the steps of analysis. Especially, the quality of isolated nucleic acids (RNA or DNA) is crucial for gene expression analysis^3^. Hence, the research of improved nucleic acid extraction is indispensable.

Nowadays, many extraction methods for DNA or RNA have been developed for commercial uses. These extraction methods can be roughly divided into two types: liquid phase extraction and solid phase extraction. The method of liquid extraction is to use organic solvents and salts to precipitate and separate RNA from the liquid phase. Phenol–chloroform extraction method developed by Chomczynski and Sacchi^4^ for RNA isolation utilizes 4 M guanidinium thiocyanate as the denaturant to break the cells and denature RNases and other biomolecules except for nucleic acids. The acid guanidinium thiocyanate-phenol–chloroform (AGPC) method of RNA extraction is widely used in the RNA isolation^4–6^. The concentration of guanidinium also has an important influence on the phase partitioning of nucleic acids into the phenol phase or the silica solid phase, which is the critical answer to explain the efficiency problem of small RNA isolation by using silica-based RNA isolation kits^7^.

Solid phase extraction methods are based on the interactions between the functional groups of nucleic acids and solid sorbents under particular conditions. Since 1990, Boom et al.^8^ developed the use of silica particles to adsorb nucleic acids, and most of the commercially available nucleic acid extraction kits currently use silica as the solid-phase sorbent. The adsorption of nucleic acids on the silica surface can be regulated with the use of chaotropic agent at different pH and concentration. Guanidinium is the most popular chaotropic agent used in many current commercial RNA extraction kits, and nucleic acids may partition into the phenol phase or onto the silica solid phase in the presence of guanidinium^7^. The binding between nucleic acids and silica surfaces depends on the intermolecular electrostatic interactions, hydrogen bond and the dehydration of the silica surface^9^. The presence of monovalent or divalent ions, pH and/or ionic strength in the media, and temperature are all the influence factors on the interaction of the nucleic acid with the silica surface. The presence of Ca^2+^ in solutions is found that it greatly enhances the deposition behavior of RNA on silica surfaces^10^, and the efficiency of divalent cations (Ca^2+^ and Mg^2+^) is higher than Na^+^ in promoting adsorption^11^. These positively charged ions can promote the formation of salt bridges between the negatively charged silica surface and the backbone of nucleic acids at high salt conditions. Franchi et al.^11^ found that divalent cations (Ca^2+^ and Mg^2+^ > 1 mM) were more efficient than monovalent cations (Na^+^  > 10 mM) in mediating the adsorption of nucleic acids on the negatively charged clay minerals. Shen et al.^10^ investigated the RNA deposition on bare silica surfaces was examined over a wide range of ionic strength in both NaCl (5–100 mM) and CaCl_2_ (0.5–5 mM) at pH 6.0 and pH 8.0. They found that increasing solution ionic strength in both NaCl and CaCl_2_ at both pH conditions would lead to greater deposition efficiencies of RNA. At the same ionic strength (5 mM), Ca^2+^ would bind to RNA molecules was greater than that in NaCl solutions. Other positively charged silica materials, like the silica surfaces modified with aminosilane and chitosan, are to utilize the positive charges produced by the protonation of amino to interact with the negatively charged nucleic acids^12^. In another study, we had been investigated the adsorption mechanisms of DNA with mesoporous silica particles through thermodynamics. We found that the exothermic enthalpy mechanism for GuHCl or GuSCN could form more salt bridges and shelve the electrostatic repulsion in high salt concentration (1 M) at pH 9, and the chaotropic salts could increase the adsorption amount compared with the kosmotropic salts. In this study, the main focus is on the mechanism involved the affinity between negatively charged nucleic acid and negatively charged silica surface.

Many commercially available solid-phase nucleic acid extraction kits use the solvent of low dielectric constant, like ethanol or isopropanol, before pipetting into the silica column for reducing the polarity of the aqueous solution. Besides, the high-concentration chaotropic agent, such as guanidine thiocyanate, is also simultaneously added to neutralize electricity and produce the effect of dehydration in order to promote the adsorption of nucleic acids to on the silica column. In summary, the binding mechanism of nucleic acid adsorption on the silica surface mainly includes four factors: hydrogen bond, salt bridge and electrostatic force formed between the nucleic acid and the silica surface, and the solubility of nucleic acid. In the extraction of circulating miRNA, silica column-based RNA extraction methods exhibited more effective and reliable than the liquid–liquid extraction protocols^13,14^. In the study reported by Duy et al.^15^, they compared five column-based RNA purification kits on the recovery of miRNA extraction and found that the QIAGEN RNeasy, which combining liquid and solid phase RNA extraction, yielded the highest miRNA recovery among the five kits. Although there are some differences in the comparison of many extraction kits, it is worth noting that the obtained RNA recovery via extraction by combining solid phase and liquid phase extractions is relatively high and stable. Nowadays, most extraction kits used in research are based on this type of extraction kit. Although the extraction methods used in the same type of extraction kits are similar, the RNA recovery is different, which means that there still has a space for improvement in RNA extraction.

In this study, the RNA recovery of extraction was determined according to the qPCR analysis of target miRNA. The effects of Ca^2+^ and ethanol concentration on miRNA adsorption and the effects of temperature and pH of the eluate on miRNA deposition were studied, respectively. From the experiments, the optimal conditions for miRNA extraction could be discovered and applied to modify the original extraction procedure. The values of RNA recovery obtained by modified the original extraction procedures were compared. Based on experimental results, the modified extraction procedure could successfully exploit to increase the recovery of miRNA.

## Material and methods

### Chemical reagents and microRNA sequences

Tris–EDTA (TE) buffer solution (Tris–HCl 0.2 M, EDTA 0.02 M, pH 8.0) was obtained from Hycell international Co., Ltd (Taiwan). Ethanol (99.5%), calcium chloride and chloroform were purchased from Echo Chemical Co., Ltd (Taiwan). Ultrapure distilled water was from Invitrogen (USA) and deionized water was purified by an ultra-pure water system (18.2 MΩ·cm, Millipore, USA). Two microRNA sequences were synthesized by MDBio Inc. (Taiwan). The sequence of miR-39 was 5′-UCACCGGGUGUAAAUCAGCUUG-3′ and the sequence of miR-21 was 5′-UAGCUUAUCAGACUGAU GUUGA-3′. The 2D and 3D structures for miR-21 and miR-39 were predicted by using RNAfold^16^ and RNAComposer^17^ websites (as shown in Figs. S1 and S2). These single-stranded RNAs still had the tendency for forming the secondary structures, and paired and unpaired nucleotides existed in the structures. By using the basic melting temperature calculation provided by OligoCalc^18^, the melting temperatures for miR-21 and miR-39 are 49.2 °C and 54.8 °C, respectively. The miRNeasy Mini Kit (50) (QIAGEN, Germany) was used for the purification of microRNA and total RNA from cultured cells, and QIAzol Lysis Reagent (phenol/guanidine-based solution) was also brought from QIAGEN (Germany). Taqman® MicroRNA Reverse Transcription Kit and TaqMan® Small RNA Assays were brought from Life Technologies (USA). PBS (phosphate buffered saline) was obtained from Thermo Fisher Scientific Inc. (USA). All other chemicals used in this study were reagent grade.

### Cell culture and standard microRNA extraction

**Figure 1:**
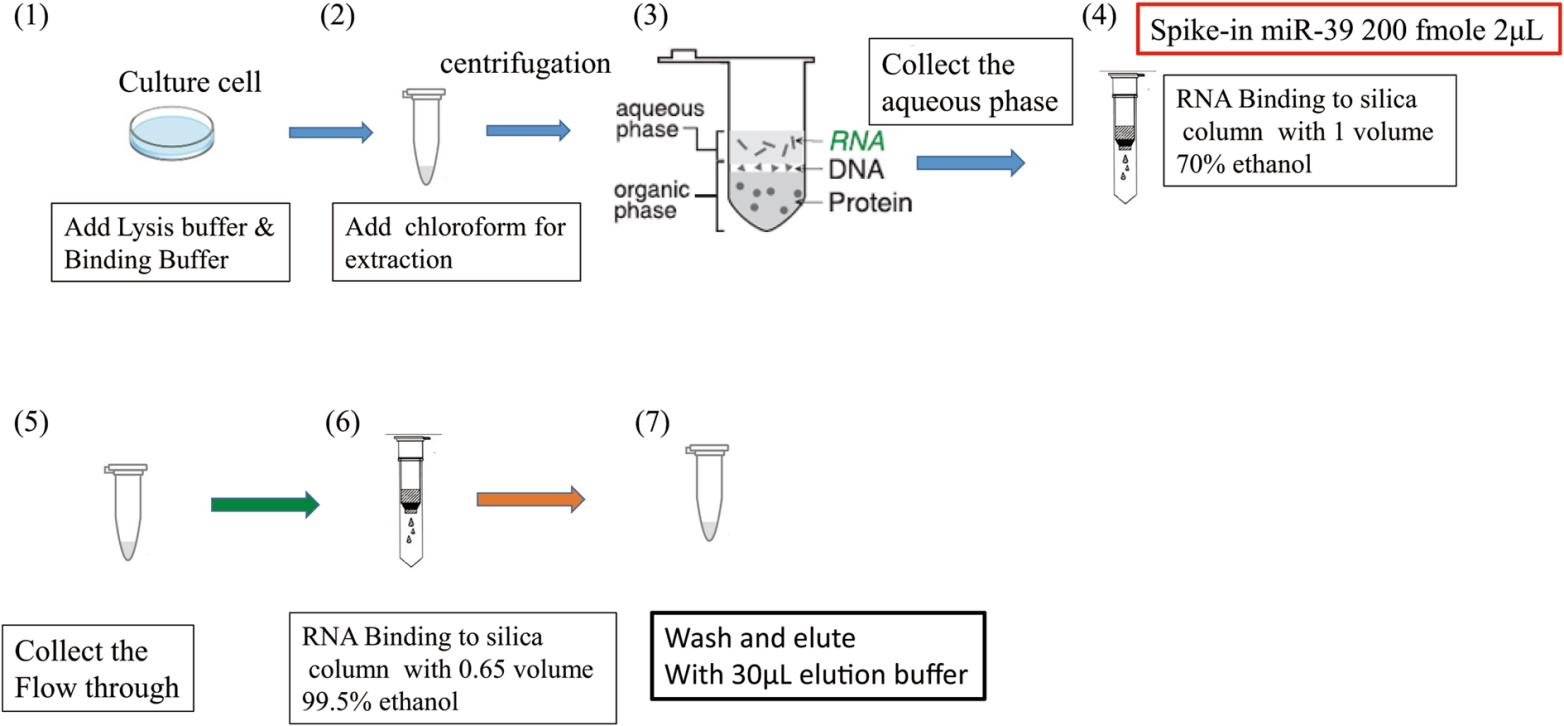
Schematic illustration of extraction steps of miRNA-enriched total RNA.

The source of HCT 116 human colon cancer cells was supplied by the lab of Prof. Li-Jen Su at the Department of Biomedical Sciences & Engineering of National Central University, Taiwan. The whole extraction steps of miRNA-enriched total RNA are shown in Fig. 1. The detailed descriptions about the extraction procedure present in the Supplementary Information.

### Modified conditions for miRNA isolation

In order to get optimized extraction results of miRNA, we herein adopted three concentrations of ethanol solutions (60%, 65%, and 70% (v/v)) in the adsorption process of miRNA isolation. The solubility of small fragments of RNA and dehydration can be reduced and enhanced, respectively, by increasing the alcohol concentration during adsorption^12^. In addition, Shen's research^10^ indicates that the addition of divalent calcium ions is very helpful for the adsorption of nucleic acids on the surface of silica. The reason is that divalent cations have better charge shielding ability and can connect the negative charge of the phosphate backbone on the nucleic acid with the deprotonated silanol group on the surface of silica to form a salt bridge, thereby increasing the amount of RNA adsorbed on the surface of silica. Hence, we also tried to add calcium chloride in the adsorbing on the silica column, which was expected to increase miRNA recovery. The scheme of improving the adsorption process of miRNA isolation is presented in Fig. S3.

For the elution process of miRNA isolation, we changed the pH or temperature of the eluent to improve the desorption efficiency of the RNA adsorbed on the silica column to find the best conditions (the conception as shown in Fig. S4). In Smerkova's study^19^, they find that the amount of eluted DNA from the silica magnetic particles increases as increasing the temperature or pH of elution solution. Increasing pH can raise the amount of negative charge on the surface of silica, leading to the increment of electrostatic repulsion force with nucleic acids. As for increasing the temperature of the elution solution, a higher temperature can break the hydrogen bond between the nucleic acid and the surface of silicon dioxide, so that the nucleic acid can be detached and washed down more easily.

These experimental design parameters of adsorption and elution have been confirmed for large fragments of nucleic acids, but these conditions are not yet known for miRNA extraction applications. Therefore, we anticipated following the same rules to find an optimized miRNA isolation method for increasing the efficiency of small fragments of nucleic acid recovery.

### Reverse transcription and quantitative real-time PCR

The StepOnePlusTM Real-Time PCR (Applied Biosystems, USA) was adopted to perform reverse transcription (RT) and qPCR experiments. In the qPCR analysis of miRNA, the experiments related to changing the extraction parameters were to use three replicates of the cells cultured at the same time for biological replication, and three replicates were used in the quantitative fluorescence analysis of qPCR for technical replicates. To amplify the miRNA obtained from cells, the TaqMan MicroRNA Reverse Transcription kit was unfrozen on ice before use. A PCR master mix was prepared by following the instructions, and the volume of master mix for each reaction is 7 μl, containing 0.15 μl of 100 mM dNTPs, 1 μl of reverse transcriptase (50 U/μl), 1.5 μl of 10X reverse transcription buffer, 0.19 μl of RNase inhibitor (20 U/μl) and 4.16 μl of nuclease-free water. The nucleic acid concentration was determined according to the absorbance of the miRNA sample in the measurement of a UV/Vis spectrophotometer. For each RT reaction, a total volume of 15 μl was composed of 5 μl of miRNA sample, 7 μl of master mix, and 3 μl of the reverse transcription primer provided with the kit The solution for the mixture solution was mixed uniformly, and let it stand on ice for 5 min prior to performing PCR. The reaction conditions were followed by 30 min at 16 °C, 30 min at 42 °C, 5 min at 85 °C, and final cooled down to 4 °C.

For the qPCR amplification, the reagents of TaqMan® Small RNA Assays were unfrozen on ice and then gently shake to mix evenly. The reactions were carried out in triplicate in a total volume of 20 μl, and each reaction contained 10 μl of TaqMan® Universal Master Mix II, no UNG, 1.33 μl of product from RT reaction, 7.67 μl of nuclease-free water and 1 μl of TaqMan® Small RNA Assay (20X). The number of experiments for each reaction is at least 2–3 times, so the amount of data points obtained for each reaction is around 6 to 9. According to the instructions, the thermal-cycling conditions for qPCR were programmed as follows: 10 min at 95 °C for enzyme activation, and the reactions were run for 40 cycles at 95 °C for 15 s and at 60 °C for 60 s. After the setup, the plate was loaded into the real-time PCR instrument to run the plate.

### Statistical analysis

The SPSS statistical software (version 18.0) was utilized to perform the statistical analysis. For the comparison of differences in Ct values for miR-21 and miR-39 groups, the data were estimated by using one-way ANOVA with post-hoc Tukey Test. For comparing the data of one kind of miRNA under 2 operating conditions, we just used one-way ANOVA for evaluation of difference in Ct values. The cut-off values for statistical significance were set at *p* < 0.05, *p* < 0.01 and *p* < 0.001, which denoted as *, **, and ***, respectively.

## Results and discussion

### The improvement of the elution process of miRNA isolation

Hydrogen bonds, van der Waals forces and hydrophobic interactions, electrostatic and salt bridge interactions are the main forces to effect the interactions between nucleic acids and silica surface. To improve the elution process of miRNA isolation, the temperature or the pH of the eluting solution was raised. Rising the temperature in the elution process can break the hydrogen bond between the RNA and silica surface to decrease the hydrogen bonding forming. As for raising the pH value of eluent, it can enhance the electrostatic repulsive force between the RNA and silica surface to let the nucleic acid desorb on the surface of silicon dioxide. First, we changed the temperature of RNase free water used in the final step of miRNA extraction to 55 °C. The main reason of selecting 55 °C as the elution temperature from binding mechanism perspective is that the elution/desorption of miRNA from silica surface at pH ~ 9 and low or no salt is facilitated by the electrostatic repulsive forces, so the higher temperature is to promote the kinetic energy of the adsorbate (miRNA) to be eluted out of the adsorbent (silica). Furthermore, by using the basic melting temperature calculation provided by OligoCalc^18^, the melting temperatures for miR-21 and miR-39 are 49.2 °C and 54.8 °C, respectively.

The silica membrane spin column with the collection tube was placed in a dry bath, the temperature was set to 55 °C, and the eluent was added to let it stand for 5 min and then centrifuged. Quantitative PCR was utilized to measure the extraction efficiency of exogenous miR-39 and the expression of endogenous miR-21. The Ct (or threshold cycle) value was adopted as a standard to determine the expression level of the target gene. When the intensity of fluorescence increases due to the amplification of the qPCR target gene template, the fluorescence of the PCR product can be detected because the fluorescence signal exceeds the background signal. The cycle number when the fluorescence of a PCR product reaches the threshold is the Ct value. Therefore, a smaller Ct value means the greater the concentration of the starting template, and vice versa.

**Figure 2:**
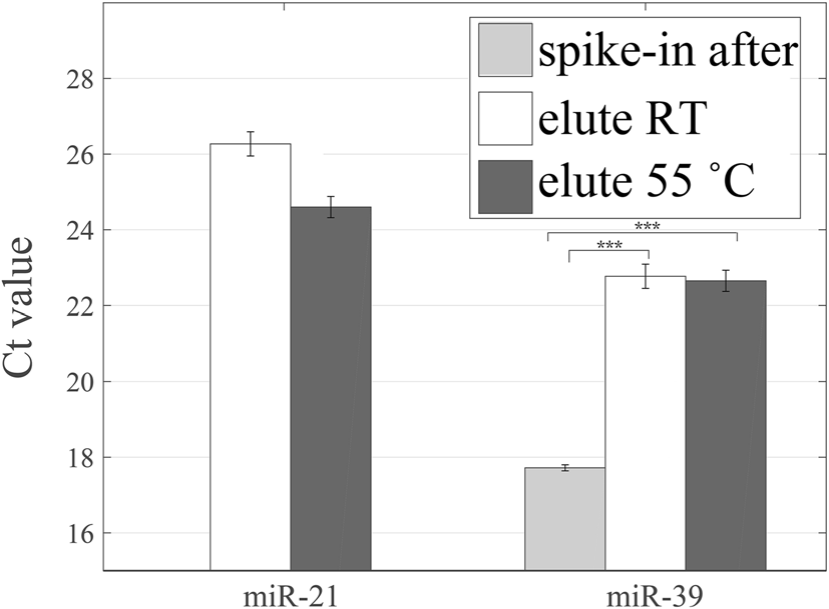
The Ct values for the extraction of miR-21 and miR-39 by using the elution temperatures at the room temperature or 55 °C. The expression of spike-in exogenous miR-39 eluting at room temperature is also compared. (***, *p* < 0.001).

Figure 2 (data shown in Supplementary Table S2) shows the effect of elution temperature on the extractions of miR-21 and miR-39. The spike-in exogenous miR-39 with the concentration of 200 fM was added in the adsorption process (as shown in Fig. 1). In order to know the recovery of miR-39, the exogenous miR-39 (200 fM) was added to the eluted RNA after the extraction step that was called as spike-in after extraction. For miR-39, statistical results showed that Ct values of extraction at room temperature and 55 °C reached statistically significant differences compared with the Ct values of spike-in after extraction. The statistical results indicated that a lot amount of miRNA lost in the extraction process. As for the endogenous miR-21, the Ct value became about 1.6 less than that eluting at room temperature when the elution temperature reached 55 °C. However, the increase of elution temperature has less effect on the extraction of miR-39. The Ct value of miR-39 eluting at 55 °C is about 0.1 less than that of miR-39 eluting at room temperature. In theory, the rising temperature can destroy the hydrogen bonding between residual miRNAs and the silica surface for improving the desorption efficiency. Basically, the temperature at 55 °C is above the melting temperatures of miR-21 and miR-39, leading to the secondary structures of miRNAs that will dissociate to become single-stranded structures. Hence, more unpaired nucleotides will interact with the charges on the silica surface, which should increase the repulsion electrostatic forces between the miRNAs and the silica surface, and the elution efficiency. According to Smerkova's research^19^, DNA yield increased with the rising elution temperature, and they obtained the highest amount of eluted DNA at 99 °C. The DNA yield obtained at 50 °C was also greater than that obtained at 20 °C or 35 °C. Our results indicated that raising temperature could slightly decrease the Ct values for miR-21 and miR-39. Based on the results, we think that raising temperature can promote the desorption efficiency of miRNA in keeping with the theory and the findings from other researches.

**Figure 3:**
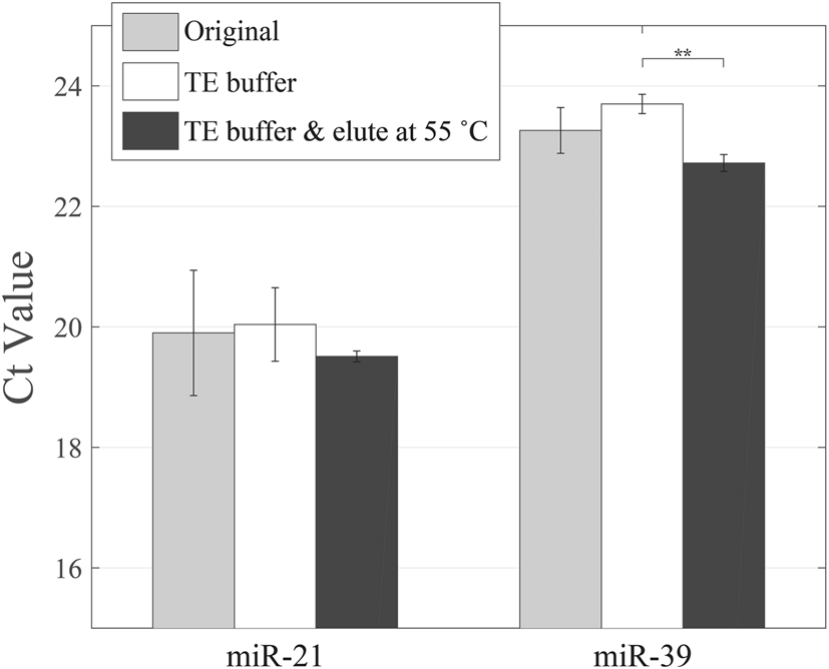
Improvement of the elution process of miRNA isolation by changing the pH value and/or temperature of elution buffer. The higher pH value and temperature at the elution process enhance the electrostatic repulsion and interrupting the binding between the miRNA and silica column. (**, *p* < 0.01).

It is known from the literature that increasing pH conditions of elution solutions can cause the higher desorption efficiency^19^. Exposing to water, the silica surface typically exhibits siloxane (Si–O–Si), silanol (Si–OH), and silanolate (Si–O−) groups^20^. For the silanolate groups, the pKa value ranging from 2 to 3, resulting partial deprotonation of the silica surface under most common conditions (at pH above pKa value). Besides, the silica surface comprises two types of Si–OH groups, and the pKa values of isolated and germinal silanol groups are 4.5 and 8.5, respectively^21^. Therefore, increasing pH of elution solution can increase the number of deprotonated silanol groups and rise the repulsion force between nucleic acids and the silica surface in theory (miRNA: negatively charged phosphate groups; silica surface: high negative charge density). On the contrary, lowing the solution pH reduces the negative charge density of silica surface. In general, the ions in solution can cause the screening effect of reducing the repulsion force and the formation of salt bridge between nucleic acids and the silica surface, and therefore RNase free water is usually utilized in the elution step. Herein, TE buffer (Tris–HCl 20 mM, EDTA 2 mM, pH 8.0) solution was used to replace the original elution solution (RNase free water). The Ct values obtained from using original elution solution and TE buffer at room temperature, and TE buffer at 55 °C were compared in the experiments. Experimental results showed that the Ct value for miR-21 was slightly increased by about 0.1 and that for miR-39 was increased by about 0.5 compared with the results by using the original elute solution (Fig. 3 and Table S3). While using TE buffer and raising the elution temperature to 55 °C, it was worth noting that the Ct values of miR-21 and miR-39 were lower than the original extraction method by about 0.4 and 0.5, respectively. This consequences indicated the use of TE buffer and increasing the temperature will improve the recovery of miR-21 and miR-39. Nevertheless, statistical results only showed that the groups between using TE buffer and using TE buffer at 55 °C had statistical significant. TE buffer contains EDTA may be a concern to influence the qPCR reaction, however, the concentration of EDTA below 0.5 mM could not cause any effect to the qPCR reaction^22^. In our study, the concentration of EDTA in the mixed solution for the qPCR reaction is estimated below 1 × 10^–5^ M.

### The improvement of the adsorption process of miRNA isolation

Calcium ions have been shown to be significantly helpful for enhancing the adsorption of nucleic acids on silica surfaces^10,11^. The presence of divalent cations (Ca^2+^) in solutions can intercalate between the phosphate groups of polynucleotides and the negatively charged inorganic substrate, forming the salt bridge, which provides a strong electrostatic attraction of nucleic acids and silica surfaces^11^. The effect of divalent cations is better than that of monovalent cations. In our research, calcium chloride solution was added at step 4 shown in Fig. 1 (as the step 7 listed in Table S1) during the small RNA adsorbed to the silica column and make the overall calcium ion concentration is at 5 mM. For the adsorption, the silica surface carries low-surface negative-charge densities and miRNA is also negative charged according to the adsorption condition at pH 5.5. Therefore, the formation of salt bridge, screening effect caused by the ionic strength, and the dehydration produced by ethanol are crucial for the adsorption. As follow the conditions mentioned in the last section, the elution buffer and temperature adopted for the elution are TE buffer and 55 °C, respectively. The effect of Ca^2+^ in promoting the final recovery of miRNA is evaluated according to the Ct value.

**Figure 4:**
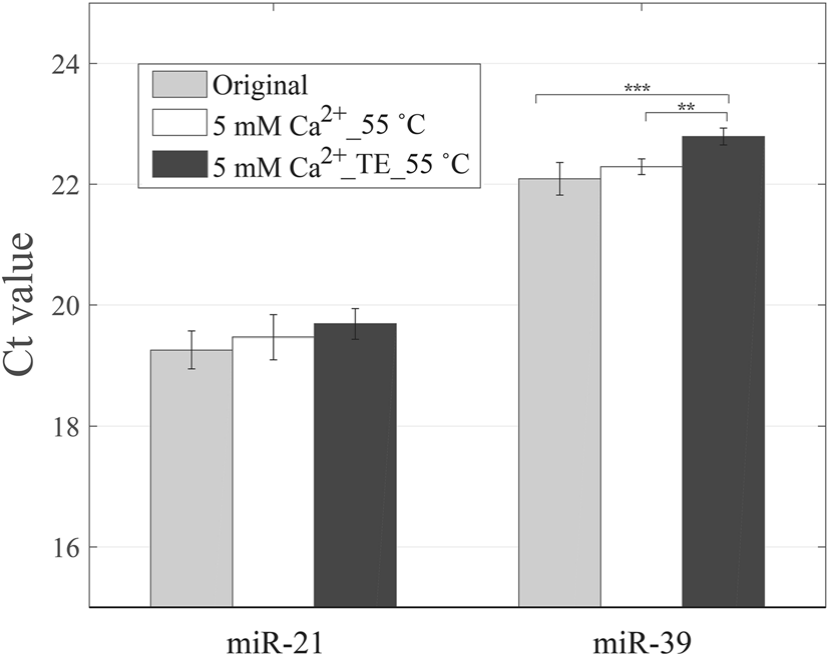
Improvement of the adsorption process of miRNA isolation by adding the CaCl_2_ and elution at 55 °C without/with TE buffer. (**, *p* < 0.01; ***, *p* < 0.001).

The results showed that the addition of divalent cations did not reduce the Ct values for miR-21 and miR-39 (Fig. 4 and Table S4). From the results, the recovery of miRNA decreased, indicating that the addition of calcium ions did not cause a positive effect on the miRNA recovery of small fragments. The statistical results even showed that the addition of Ca^2+^ could not bring promotive effect on the miRNA isolation. The original concentration of guanidine isothiocyanate in the QIAzol Lysis Reagent of RNA extraction kit is 4 M, and the final concentration of guanidine isothiocyanate used in the adsorption of nucleic acids is around 1 M due to the addition of ethanol solution. By using the pH meter, the pH value of the washing buffer containing the QIAzol Lysis Reagent and ethanol was measured as 5.5. Therefore, the negative charges formed on the silica surfaces were less and could not produce a sufficient number of salt bridges with miRNAs. In addition, we suggested that the divalent cations could counterbalance the negative charges presented in nucleic acids, and excessive cations occupied the phosphoric acids on the nucleic acid backbone and the hydroxyl groups on the surface of the silica dioxide to interfere the formation of the hydrogen bond. Compared with large fragments of RNAs, we think the addition of divalent cations has a greater impact on the extraction of small fragments of miRNAs. Because miRNA carries less negative charges than RNA and are more sensitive to the electrically neutralized effect. The above-mentioned experimental results indicated that the addition of divalent cations was unable to promote the recovery in the extraction of miRNA.

**Figure 5:**
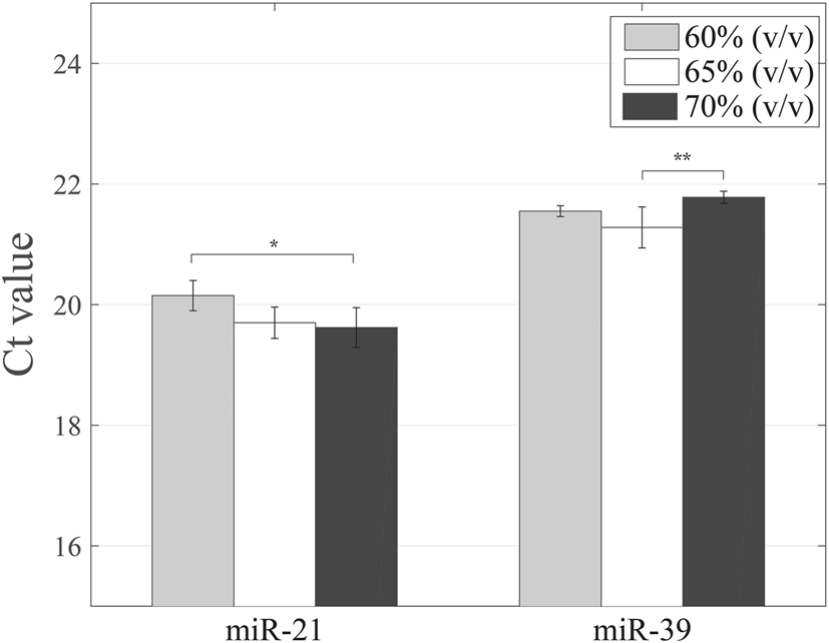
Improvement of the adsorption process of miRNA isolation with different volume concentrations of ethanol solutions. The solution with a higher ethanol concentration should decrease the solubility of miRNA and have a dehydration effect on the miRNA and silica surface. (*, *p* < 0.05; **, *p* < 0.01).

During the nucleic acid adsorbs on the silica surface, the addition of ethanol or isopropanol can reduce the solubility of nucleic acid and increase dehydration, so that the nucleic acid can be more easily precipitated and adsorbed on the surface of silica in the adsorption processes. Xu et al.^7^ found that the interphase between the water–phenol phases would vanish when the guanidinium concentrations were beyond 4 M in the aqueous phase. In our study, the function of guanidine isothiocyanate is to partition most RNAs into the aqueous phase and then isolate RNAs by the addition of the ethanol solution. Three concentrations of ethanol solutions (60%, 65%, and 70% (v/v)) were used during the miRNA adsorbed on the silica column to investigate the effect of ethanol concentration on the difference in the recovery of miRNA. For miR-21, the Ct values decreased by about 0.4 and 0.5, respectively, after changing the ethanol concentration from 60% (v/v) to 65% (v/v) and 70% (v/v) (Fig. 5 and Table S5). The difference in the Ct value for using 65% and 70% ethanol solution was slight (no statistical difference). As for miR-39, the minimal Ct value appeared when using the 65% ethanol solution in the adsorption process, and this group had a statistically significant difference compared with the group of using the 70% ethanol solution. These experimental results for both miRNAs revealed that the selection of 65% volume concentration of ethanol in the adsorption process could be the best choice to increase the miRNA recovery.

### Optimal conditions for extraction of nucleic acid

**Figure 6:**
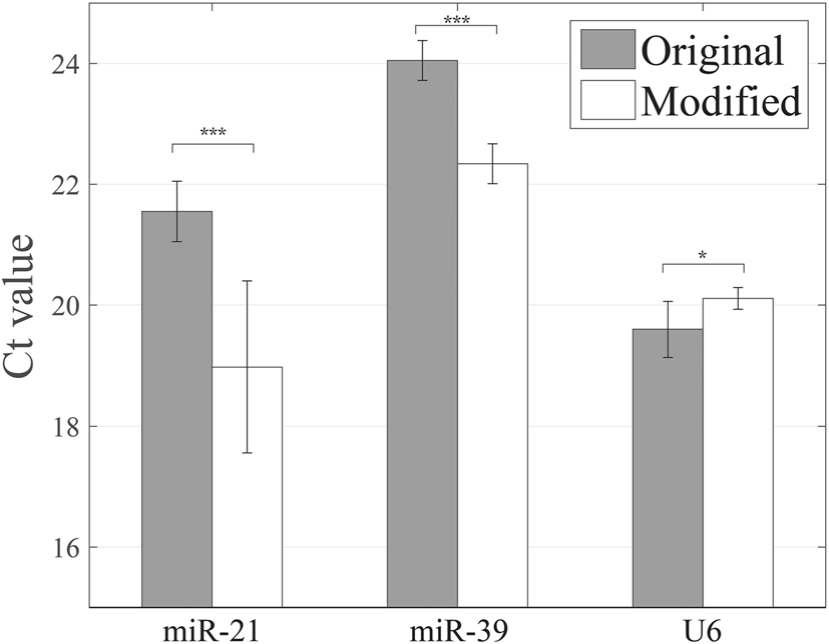
The extraction results obtained by the modified protocol compared to that of the original protocol. The modified protocol decreases the Ct values of the miR-21 and miR-39. (*, *p* < 0.05; ***, *p* < 0.001).

According to above mentioned experimental results, the optimal condition for adsorption process is using 65% volume concentration of ethanol, and the optimal conditions for elution are using TE buffer and performing elution at 55 °C. By using the optimal conditions simultaneously for the extraction of miRNA, we utilized qPCR to measure the endogenous miR-21 and U6, and exogenous miR-39 for checking whether the improved extraction procedure could increase the recovery of miRNA. U6 is a kind of small nuclear RNA (snRNA), the fragment size is about 109 nt, and is one of most commonly used internal control gene in miRNA RT‑qPCR assays^23^. U6 is a good reference gene (housekeeping gene), hence it is usually used in the study of various tissue samples and cell lines^24^. Compared with the results obtained by using original extraction procedure, Ct values for miR-21 and miR-39 decreased significantly by approximately 2.6 and 1.7, respectively, with the use of modified extraction conditions (*p* < 0.001, Fig. 6 and Table S6). By using the modified extraction procedure, the average Ct value for miR-21 was 19 with a standard deviation of 1.4, and the average and standard deviation of Ct values of miR-39 were 22.3 and 0.3. Overall, the Ct values of the two miRNA had declined, indicating that the recovery of miRNA had increased. The average Ct values of U6 in two extraction procedures are very close (original: 19.6; modified: 20.11), however, the statistical result revealed a significant difference between these two groups (*p* < 0.05). Based on these experimental result, we think that the combination of optimal conditions for the isolation of miRNA indeed promotes the recovery of miRNA. Nevertheless, the major limitation for implementation of modified extraction protocol with these optimal conditions is that the elution process needs to be performed at 55 °C.

From a thermodynamic point of view, the adsorption and desorption behavior of RNA on the silica surface can be elucidated via Gibbs free energy at a constant pressure, and the defining equation is written as Eq. (1).

1 $$\Delta \mathrm{G}=\Delta H-T\Delta \mathrm{S}$$

where ΔG is Gibbs free energy, ΔH is enthalpy, and ΔS is entropy. If ΔG is negative, the reaction is spontaneous. The buffer with pH 5.5 used in the adsorption of RNA molecule on the silica surface contains the chaotropic agent (in QIAzol Lysis Reagent) and ethanol, and the partial factors affect the adsorption of the RNA molecule are the formation of hydrogen bond and the salt bridge. The formation of hydrogen bond and the salt bridge is an exergonic process, and therefore ΔH less than 0. But, the binding is just a slightly exothermic reaction, which leads to the adsorption with little or no enthalpic contribution^9^. The nucleic acid adsorption process is also driven by an increase in entropy (ΔS) in the system that accompanies the water molecules released from the nucleic acids and silica surfaces^9,25^. Therefore, the increment of ethanol concentration in the miRNA extraction can increase the entropy in the system, and the value of ΔG can be more negative resulting in the miRNA more likely to adsorb on the silica surface.

To desorb the miRNA from the silica surface, hydrogen bonds and salt bridges between nucleic acids and the silica surfaces need to be broken. In the original operation steps of the nucleic acid extraction kit, the miRNA is eluted under low ionic strength conditions with RNase-free water with a pH value of 7.0. In our modified procedure, TE buffer with a pH value of 8.0 is adopted as the elution buffer, and eluting temperature increase to 55 °C. The reactions for breaking hydrogen bonds and salt bridges are endergonic (ΔH > 0), hence raising temperature can promote the desorption of miRNA. The entropy (ΔS) decreases due to the desorption of miRNA, and the Gibbs free energy of the system overall becomes larger that is favorable for the desorption of miRNA.

### Quantification of miRNA concentration by qPCR

**Figure 7:**
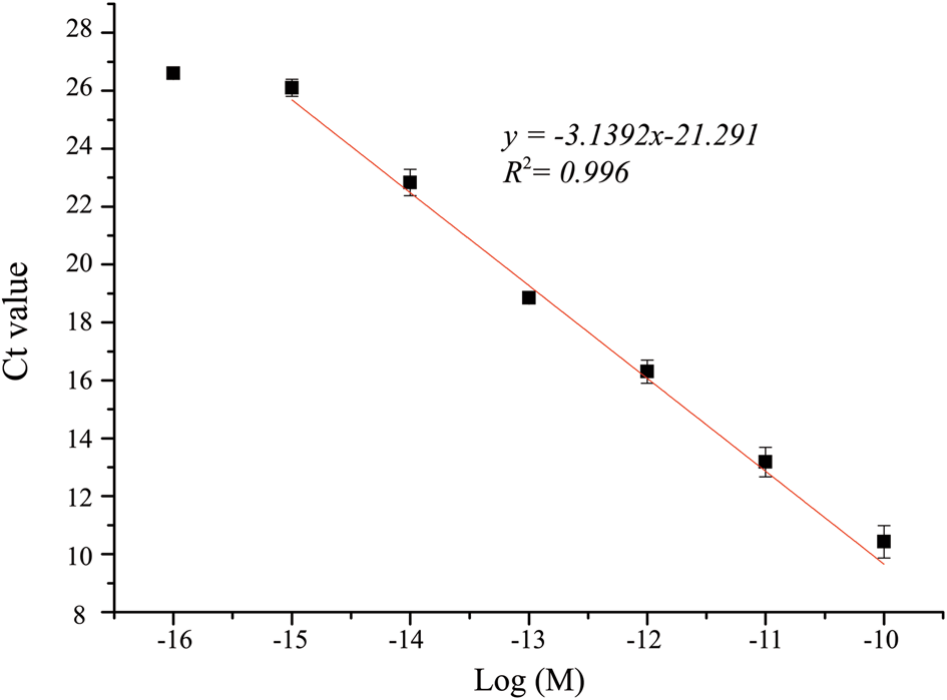
Calibration curve of the proposed extraction method. It shows the linear relationship between Ct value and logarithmic concentration of miR-21.

In order to quantify the molar concentration of miR-21 of harvested HCT 116 cell, artificially synthesized miR-21 was prepared to a known concentration of 100 pM and then made serial dilutions as follows: 10 pM, 1 pM, 0.1 pM, 0.01 pM, 1 fM, and 0.1 fM. The qPCR was utilized to analyze these solutions with known concentrations of artificially synthesized miR-21 to obtain the calibration curve. The relationship between the Ct value and the logarithmic concentration of miR-21 is plotted in Fig. 7. It is found that the Ct value has a linear dependence on the logarithmic concentration of miR-21 in the range of 10^–10^‐10^−15^. The regression equation can be expressed as

2 $$\mathrm{y}=-3.1392\mathrm{x}-21.291 {R}^{2}=0.996$$

where y is the Ct value, x is the logarithmic concentration of miR-21, and R^2^ is the measure for the linear regression model. Using the optimal conditions for the extraction of miR-21, the Ct value is 19, and the concentration of miR-21 can be estimated as 148.6 pM according to the regression equation. The concentration of miR-21 obtained from the original extraction procedure is evaluated as 22.5 pM by using the regression equation. The proposed method shows that the concentration of miR-21 isolated by the modified protocol is about 6 times more than the original protocol.

## Conclusions

In this study, the conditions in the extraction steps of commercial kit were investigated to improve the recovery of miRNA. The modified conditions included using a 65% (v/v) solution of ethanol in the adsorption process, and using TE buffer with the pH value of 8.0 and raising the temperature to 55 °C in the elution, which had substantial enhancement on the recovery of miR-21 or spike-in miR-39. By using these optimal conditions, the improved extraction kit can increase the recovery of endogenous miR-21 by about 6 times in the light of the quantified results. Because the results of the genetic testing platform is affected by the quality and purity of the samples. From the results presented in this study, the modified extraction method can provide a higher recovery of miRNA from cells, which will be of great help for subsequent genetic testing or analysis applications.

## Supplementary information

Supplementary Information 1.
