# Supplementary material for: Improve sample preparation process for miRNA isolation from the culture cells by using silica fiber membrane
Source: Sci Rep. 2020 Dec 3;10:21132. doi: 10.1038/s41598-020-78202-8 (PMC7713297; doi:10.1038/s41598-020-78202-8)
Supplement: Supplementary file 1 — Supplementary Information 1. [file 41598_2020_78202_MOESM1_ESM.docx]

**Supplementary information**

**Improve sample preparation process for miRNA isolation from the culture cells by using silica fiber membrane**

Wen-Pin Hu^a^, Yu-Chi Chen^b^, Wen-Yih Chen^b,^*

^a^ Department of Bioinformatics and Medical Engineering, Asia University, Taichung 41354

^b^ Department of Chemical and Materials Engineering, National Central University, Jhong-Li 32001, Taiwan, Taiwan

* Corresponding author: Wen-Yih Chen

TEL:+886-3-4227151 ext.34222

FAX: +886-3-4225258

Email addresses: wychen@ncu.edu.tw (W.-Y. Chen)

Full postal address: Department of Chemical and Materials Engineering, No. 300, Zhongda Rd., Zhongli District, Taoyuan City 32001, Taiwan

**Material and Methods**

**Cell culture and standard microRNA extraction.**

The solution containing McCoy's 5A (Modified) Medium (Gibco, Cat number: 16600082) with 10% fetal bovine serum (Gibco, cat no. 10437028) and 1% penicillin-streptomycin antibiotic solution (Gibco) was adopted as the cell culture medium. All culture operations were performed in a sterile environment and cells were cultured in a cell incubator at a constant temperature of 37 °C and with 5% carbon dioxide. Initially, a culture dish (10-cm plate), which had 70-80 percentage of area covered by the cells, was taken from the incubator for transferring cells to new Petri dishes. The cell culture medium was warmed in a water bath at 37 °C for 10-15 min before use. To transfer cells, the culture medium was aspirated by using a vacuum pipette, and then 3 ml of PBS buffer was added to rinse the Petri dish slowly once. After that, the PBS buffer was pipetted, and the Petri dish was placed at the incubator for 3 min after adding 1ml 1X Trypsin-EDTA. After adding 3 ml of cell culture medium, the cell solution was pipetted to a 15 ml sterile centrifuge tube and mixed it gently several times. For calculating the cell number, 0.5 ml of the cell suspension was taken out from the centrifuge tube and placed it in a 1.5 ml eppendorf tube. The total number of cells in the sterile centrifuge tube could be evaluated according to the counting number obtained by using the cell counting plate. After centrifugation, the supernatant in the centrifuge tube was aspirated, and then an appropriate amount of cell culture medium was added to obtain the concentration for the cell suspension of 8×10^5^ cells/ml. Then, appropriate number of Petri dishes were prepared and 9 ml of culture medium was added to each plate. Each culture plate was seeded by adding 1 ml of cell suspension, and these plates were then placed in the incubator for 4 days.

After culturing HCT 116 cell line for 4 days, the number of cells per dish was about 5 × 10^6^ cells, and the number of cells increased about six times. The culture medium in the dish was removed, and then 2 ml of PBS buffer was slowly dripped to add into the dish, followed by gently shake and rinse, and the PBS buffer was removed by using a pipette controller eventually. This washing step had to be repeated twice. After that, 700 μL of the lysis reagent (phenol/guanidine-based QIAzol Lysis Reagent) was added and let the reagent evenly distributed on the dish. To collect the cells, a cell scraper was used to gently remove attached cells from the culture dish. Afterward, harvested cells and the lysis reagent was pipetted to a 2 ml microtube, and 140 μL of chloroform was added to the microtube and mixed evenly after shaking and leave it at room temperature for about 2 min. Then the microtube was centrifuged at a centrifuge at speed of 12000 xg for 15 min at 4 °C. After the centrifugation, the upper transparent layer of the supernatant in the microtube (aqueous phase (Fig. 1)) was pipetted to another microtube. Then, 2 μL of artificially synthesized exogenous miR-39 with a concentration of 200 fM was added to a microtube with a 300 μl of solution from the upper layer of the supernatant. The equal volume (302 μl) of 60% (v/v) ethanol was added to the microtube, and the mixed solution was transferred to a silica membrane spin column. The column was then spun at 10000 xg for 1 min at room temperature after transferring the mixed solution. After collecting about 500 μl of the filtrate, 325 μl (0.65 volume) of 99.5% (v/v) ethanol was added to mix evenly with the filtrate. The volume of mixture exceeded the 700 μl, therefore the liquid needed to be divided into two portions and transferred to the silica membrane spin column to centrifuge at 10000 xg for 1 min at room temperature in sequence. The filtrates were discarded and the silica membrane spin column was washed by slowly pipetting the wash buffer inside the column about 20 times. After that, the silica membrane spin column was put in a centrifuge and spun at 10000 xg for 1 min at room temperature, which needed to repeat twice. Subsequently, the cap of the silica membrane spin column was opened and centrifuged at 10000 xg for 5 min to remove the residual liquid in the column. After the step, the silica membrane spin column was put into the collection tube. 30 μl of RNase free water was added to the silica membrane spin column, and the tube was centrifuged at a speed of 10000 xg for 5 min, and finally, the miRNA-enriched total RNA could be obtained. The above-mentioned procedures are the original protocol we adopted for miRNA isolation. Table S1. shows the summary of the extraction process and the comparison between the original and modified protocols for miRNA isolation.

**
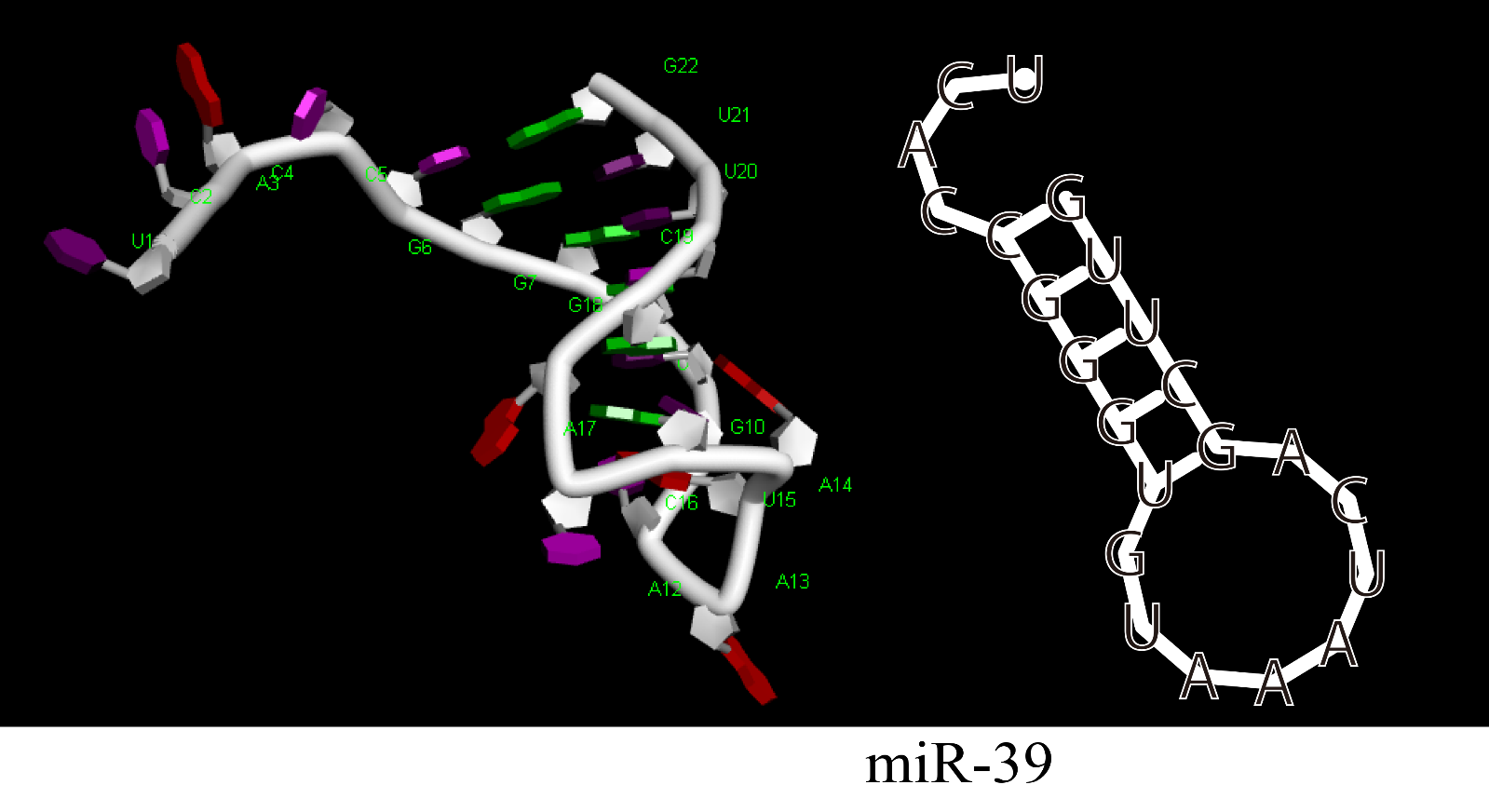
**

**Figure S1.** The 2D and 3D structures for miR-39 predicted by RNAfold and RNAComposer websites, respectively.

**
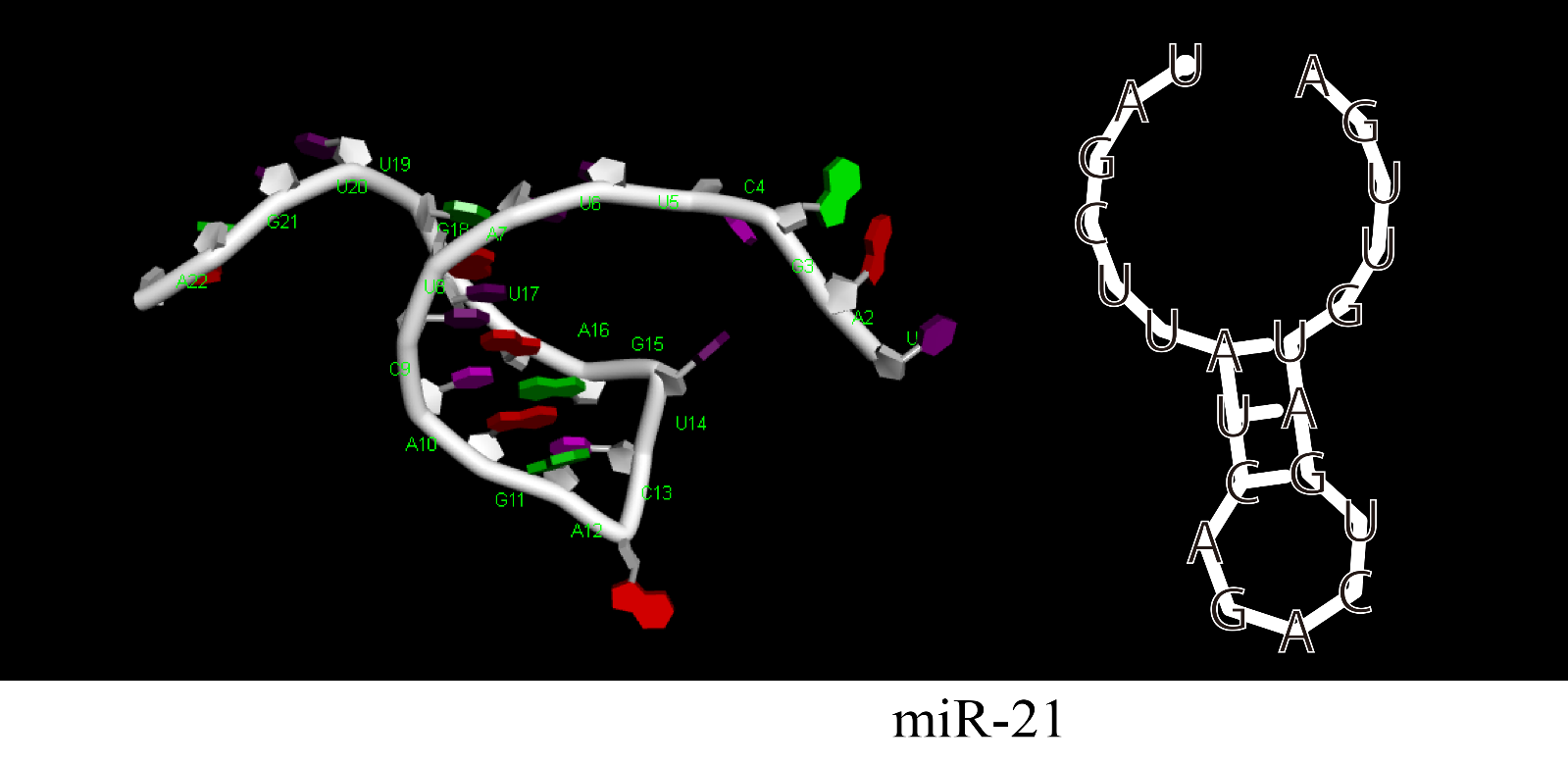
**

**Figure S2.** The 2D and 3D structures for miR-21predicted by RNAfold and RNAComposer websites, respectively.

**
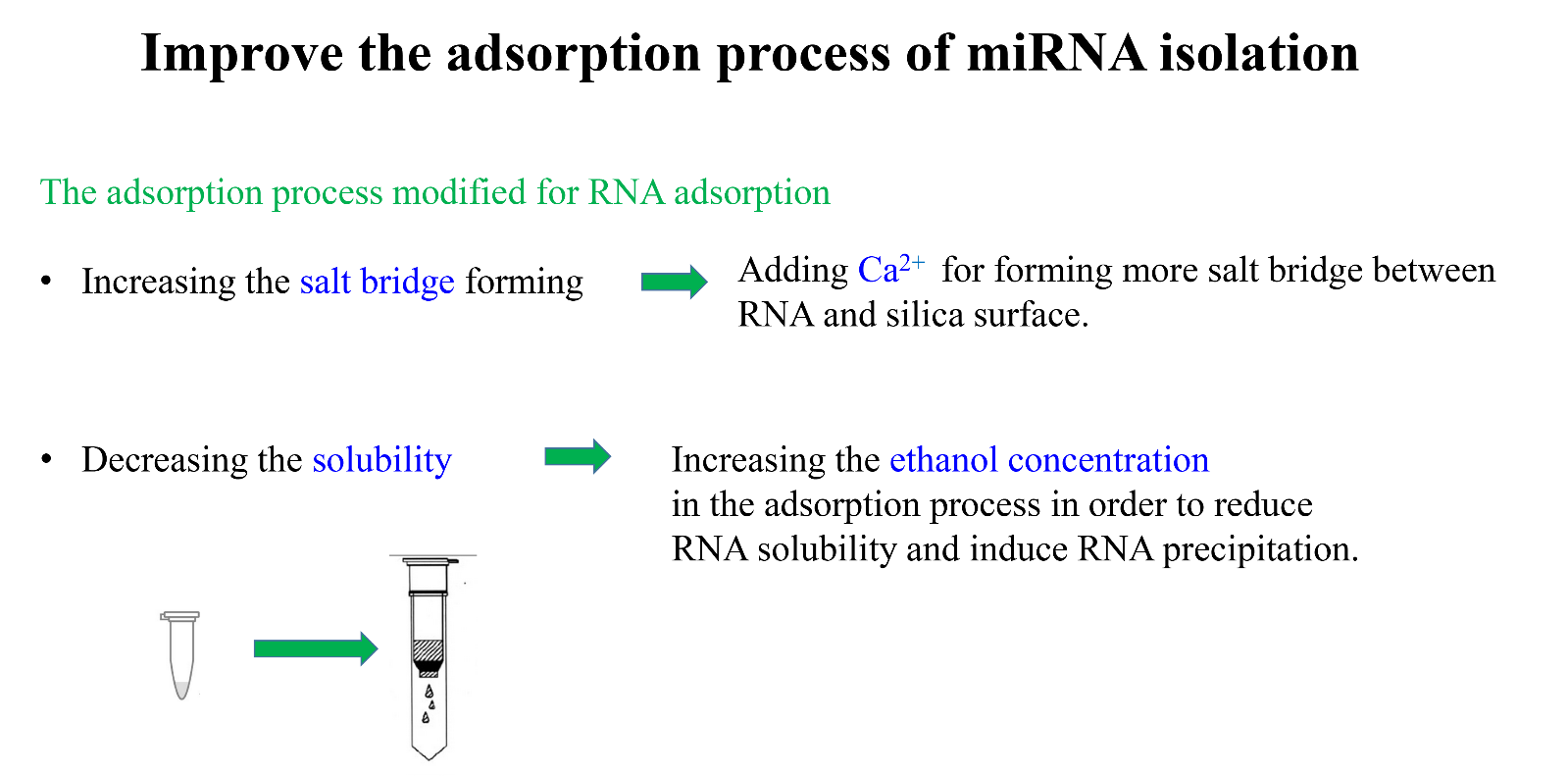
**

**Figure S3.** The scheme of improving the adsorption process of miRNA isolation

**
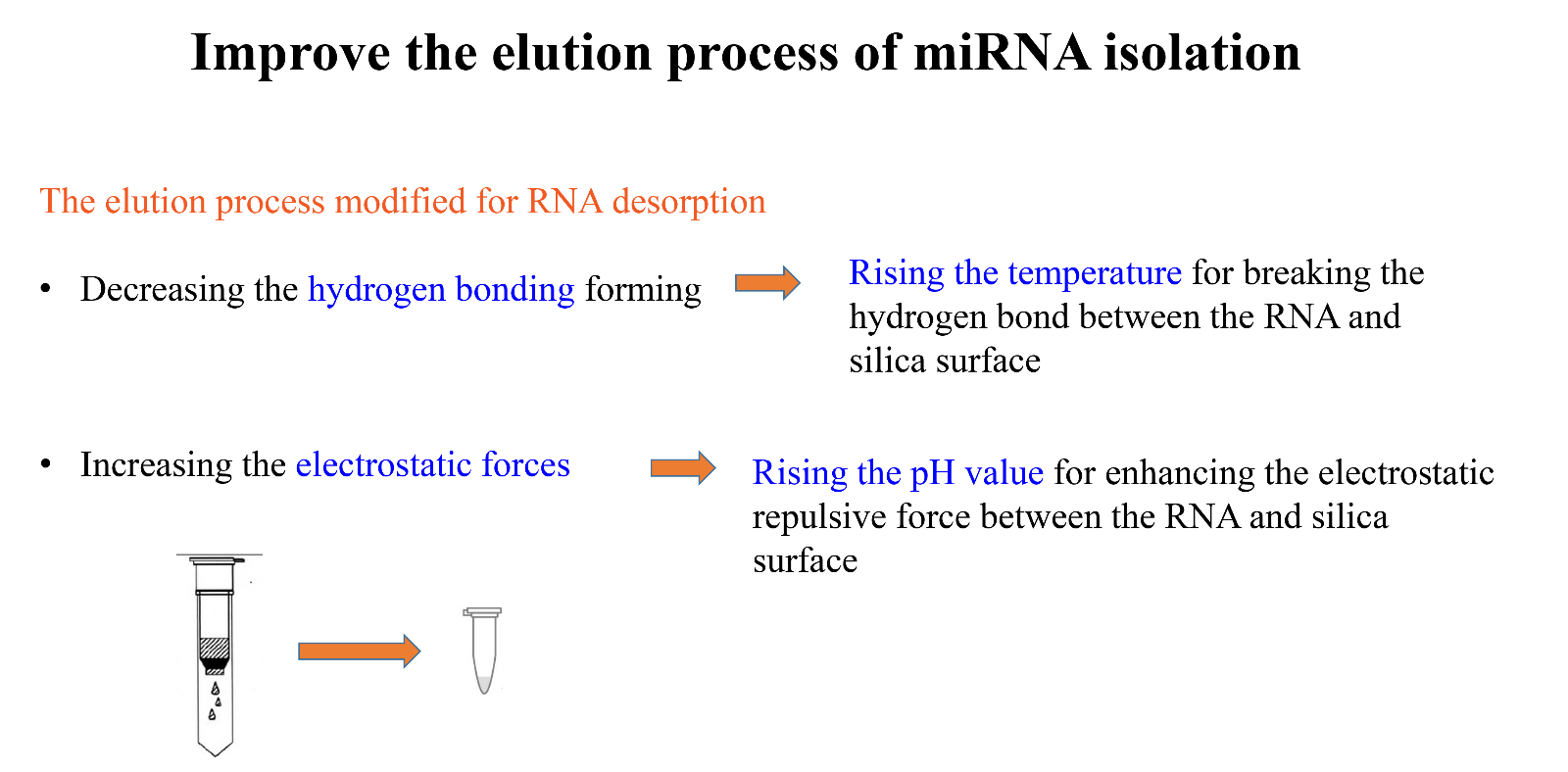
**

**Figure S4.** The scheme of improving the elution process of miRNA isolation

**Table S1.** The comparison between the original and modified protocols for miRNA isolation. The modified conditions are marked in bold.

| **Original protocol for miRNA isolation** | **Modified protocol for miRNA isolation** |
| --- | --- |
| 1. Harvest cultured cells | 1. Harvesting cultured cells |
| 2. Disrupt the cells by adding QIAzol Lysis Reagent | 2. Disrupt the cells by adding QIAzol Lysis Reagent |
| 3. Homogenize the lysate and chloroform | 3. Homogenize the lysate and chloroform |
| 4. Centrifuge the microtube and obtain the supernatant | 4. Centrifuge the microtube and obtain the supernatant |
| 5. Add the exogenous miRNA spike-in control in | 5. Add the exogenous miRNA spike-in control |
| 6. Add equal volume of 60% ethanol to the homogenized lysate | **6. Add equal volume of 65% or 70% ethanol to the homogenized lysate** |
| 7. Transfer the mixed solution (step 6) to a silica membrane spin column and centrifuge the spin column | **7. Transfer the mixed solution (step 6) to a silica membrane spin column, add calcium chloride and centrifuge the spin column*** |
| 8. Collect the filtrate and add 99.5% (v/v) ethanol | 8. Collect the filtrate and add 99.5% (v/v) ethanol |
| 9. Transfer the solution from step 8 to a silica membrane spin column and centrifuge the spin column | 9. Transfer the solution from step 8 to a silica membrane spin column and centrifuge the spin column |
| 10. Discarded the filtrate and remove the residual liquid in the column | 10. Discarded the filtrate and remove the residual liquid in the column |
| 11. Add the RNase free water to the silica membrane spin column for elution at room temperature | **11. Add the TE buffer (pH 8.0) to the silica membrane spin column for elution at 55 °C** |
| 12. Obtain the miRNA-enriched total RNA | 12. Obtain the miRNA-enriched total RNA |

*** Calcium chloride didn’t add in the optimal extraction protocol.**

**Table S2.** The Ct values of miR-21 and miR-39 obtained by changing the temperature of the elution buffer.

| **miRNA**  **Condition** | **miR-21** | **miR-39** |
| --- | --- | --- |
| Spike-in after | 26.86±0.29 | 17.72±0.08 |
| Elute RT | 26.27±0.32 | 22.77±0.32 |
| Elute 55 ℃ | 24.6±0.28 | 22.65±0.28 |

**Table S3.** The Ct values of miR-21 and miR-39 obtained by changing the TE buffer (pH 8.0) as the elution buffer with/without rising the elution temperature to 55 °C.

| **miRNA**  **Condition** | **miR-21** | **miR-39** |
| --- | --- | --- |
| Original | 19.91±1.04 | 23.26±0.38 |
| TE buffer | 20.04±0.61 | 23.73±0.16 |
| TE buffer elute at 55 ℃ | 19.51±0.09 | 22.72±0.14 |

**Table S4.** The Ct values for the presence of 5 mM Ca^2+^ in the binding solution and elution at 55 °C with/without the TE buffer.

| **miRNA**  **Condition** | **miR-21** | **miR-39** |
| --- | --- | --- |
| Original | 19.26±0.31 | 22.09±0.27 |
| 5mM Ca^2+^_55 ℃ | 19.47±0.37 | 22.29±0.13 |
| 5mM Ca^2+^_TE_55 ℃ | 19.69±0.25 | 22.79±0.14 |

**Table S5.** The acquired Ct values by adjusting ethanol concentration from the original protocol 60% (v/v) to 65% (v/v) and 70% (v/v) in the adsorption process of miRNA isolation.

| **miRNA**  **Ethanol  concentration (v/v)** | **miR-21** | **miR-39** |
| --- | --- | --- |
| 60% | 20.15±0.25 | 21.55±0.09 |
| 65% | 19.7±0.26 | 21.28±0.34 |
| 70% | 19.62±0.33 | 21.78±0.1 |

**Table S6.** The Ct values for the original and modified protocols. The modified protocol includes using the TE buffer and rising the elution temperature to 55 °C in the elution process, and using 65% (v/v) ethanol solution in the adsorption process.

| **miRNA**  **Procedure** | **miR-21** | **miR-39** | **U6** |
| --- | --- | --- | --- |
| Original | 21.55±0.5 | 24.05±0.33 | 19.6±0.46 |
| Modified | 18.98±1.42 | 22.34±0.33 | 20.11±0.18 |
